# Epithelial model-dependent host-microbe interactions in human intestinal models under anaerobic Transwell co-culture

**DOI:** 10.3389/fmicb.2026.1886960

**Published:** 2026-07-24

**Authors:** Haneol Yang, Chan-Hyeok Park, Naeun Son, Do-Yun Kim, Ye Seul Son, Won Dong Yu, Ki-Hyun Kim, Mi-Young Son, Doo-Sang Park

**Affiliations:** 1 Korean Collection for Type Cultures (KCTC), Korea Research Institute of Bioscience and Biotechnology (KRIBB), Jeongeup, Republic of Korea; 2 Department of Biomedical Sciences, Yonsei University College of Medicine, Seoul, Republic of Korea; 3 Stem Cell Convergence Research Center, Korea Research Institute of Bioscience and Biotechnology (KRIBB), Daejeon, Republic of Korea; 4 KRIBB School of Bioscience, Korea University of Science and Technology (UST), Daejeon, Republic of Korea; 5 School of Medicine, Sungkyunkwan University, Suwon, Republic of Korea

**Keywords:** anaerobic co-culture, gut microbiota, host-microbe interactions, intestinal epithelial models, intestinal epithelium

## Abstract

The intestinal epithelium and gut microbiota exist at a dynamic aerobic-anaerobic interface that is difficult to reproduce *in vitro* because epithelial cells require oxygen, whereas many gut bacteria are oxygen-sensitive. Here, we used an accessible anaerobic Transwell co-culture framework to systematically compare epithelial model-dependent host-microbe interaction outcomes, rather than positioning the system as a fundamentally new device. Using this platform, we compared the widely used tumor-derived Caco-2 cells with human intestinal epithelial cells (hIECs) derived from pluripotent stem cells to evaluate how epithelial cell origin influences microbial growth, metabolite dynamics, and host responses. Across multiple representative bacterial species, epithelial model choice strongly affected co-culture outcomes. When interpreted relative to each model's corresponding medium-only control, hIEC-based cultures tended to better support the growth or persistence of *Lactiplantibacillus plantarum, Bifidobacterium longum*, and *Akkermansia muciniphila*, whereas Caco-2-based cultures showed stronger growth responses for *Escherichia coli* and *Clostridioides difficile*. Distinct differences in short-chain fatty acid production and compartmental distribution further suggested that metabolite readouts depend on epithelial model context. Microbial co-culture increased expression of intestinal transcription factors, stem cell-associated genes, and *ZO-1* in Caco-2 cells, whereas hIECs exhibited distinct transcriptional responses. Notably, under the defined hIEC culture conditions, exposure to the opportunistic pathogen *E. coli* was associated with increased expression of epithelial markers related to differentiation, lineage specification, and maturation, whereas Caco-2 cells showed increased cell death. Together, these findings suggest that epithelial cell origin is an important factor shaping *in vitro* host–microbe interaction outcomes and demonstrate that side-by-side comparison under an anaerobic Transwell co-culture framework can reveal model-dependent responses that may be obscured in single-model studies.

## 1 Introduction

The gut microbiota plays a crucial role in maintaining host health through continuous interactions with the intestinal epithelium. Commensal microorganisms contribute to intestinal homeostasis by supplying essential nutrients, protecting against pathogens, and supporting mucosal immune functions (Aguilar-Rojas et al., 2020). Conversely, intestinal epithelial cells also exert regulatory control over the microbial ecosystem. The epithelium regulates microbial colonization through mucus secretion, the production of antimicrobial peptides, and epithelial signaling pathways that shape microbial diversity and composition (Soderholm and Pedicord, 2019; Okumura and Takeda, 2017). These bidirectional interactions between microbes and epithelial cells are essential for preserving epithelial barrier function and preventing dysbiosis along the mucosal surface.

Despite their close association *in vivo*, recapitulating these interactions *in vitro* remains technically challenging due to the conflicting physiological requirements of microbes and host cells. Most gut microbes are strict or obligate anaerobes, whereas intestinal epithelial cells that interact with them require oxygen for survival (Shang et al., 2025), complicating the establishment of conditions that support both simultaneously. Accordingly, a variety of *in vitro* systems have been developed to model host–microbe interactions at the intestinal surface, including Transwell platforms, microfluidic devices, and gut-on-a-chip systems (Ulluwishewa et al., 2015; Shah et al., 2016; Lee et al., 2024). Among these, Transwell-based approaches are widely used because they are experimentally accessible and compatible with conventional epithelial assays. However, the physiological relevance of Transwell-based systems depends strongly on the epithelial model used, and their application has often been limited to a narrow range of bacterial species, both of which have been insufficiently explored.

Most studies investigating microbiota–epithelium interactions have relied on tumor-derived intestinal cell lines, such as Caco-2, HT-29, and HCT-8 cells, which do not fully recapitulate the structure or physiology of the human intestine (Hares et al., 2021). These cell lines lack cellular diversity, exhibit limited mucin production, and fail to mimic the physiological characteristics of the native intestinal epithelium (Son et al., 2023). In contrast, functional human intestinal epithelial cells (hIECs) derived from human pluripotent stem cells (hPSCs) offer a physiologically relevant alternative. hIECs display key features of the human gut epithelium, including apical–basolateral polarity and the presence of multiple intestinal cell types such as Paneth cells, mucus-secreting goblet cells, enterocytes, and hormone-secreting enteroendocrine cells, although the relative abundance and maturity of each lineage may differ from native tissue. Moreover, hIECs express mucin-associated genes and tight junction components, supporting better approximation of native intestinal barrier features than conventional tumor-derived intestinal cell lines.

In this study, we hypothesized that epithelial cell origin is a major determinant of *in vitro* host-microbe interaction outcomes. To test this, we used an accessible anaerobic Transwell co-culture system and compared hIECs with the widely used Caco-2 model in co-culture with representative commensal and opportunistic bacterial species, including *Lactiplantibacillus plantarum* (*L. plantarum*), *Bifidobacterium longum* (*B. longum*), *Akkermansia muciniphila* (*A. muciniphila*), *Clostridioides difficile* (*C. difficile*), and *Escherichia coli* (*E. coli*). We assessed bacterial viability, short-chain fatty acid production, and microbe-induced changes in epithelial gene expression to determine how epithelial cell origin influences microbial behavior and host responses *in vitro*.

## 2 Materials and methods

### 2.1 Bacterial strains and culture conditions

The bacterial strains used in this study are listed in Supplementary Table 1. All bacteria were grown at 37 °C under anaerobic conditions in an anaerobic chamber (Coy Laboratory Products, Grass Lake, MI, USA) composed of N_2_ (80%), H_2_ (10%), and CO_2_ (10%). *L. plantarum* and *B. longum* were cultured in De Man, Rogosa, and Sharpe (MRS; BD Biosciences, Franklin Lakes, NJ, USA) medium, and *A. muciniphila, C. difficile*, and *E. coli* were cultured in Brain Heart Infusion (BHI; BD Biosciences) medium. Before co-culture with intestinal epithelial cells, each bacterial strain was subcultured in MRS or BHI broth for 16 h.

### 2.2 Intestinal epithelial cell culture

Caco-2 cells were obtained from the Korea Collection for Type Cultures (KCTC; Jeongeup, Korea) and were grown in Minimum essential medium (MEM; Corning, NY, USA) containing 10% (v/v) fetal bovine serum (FBS; Thermo Fisher Scientific, Waltham, MA, USA) and 1% (v/v) penicillin-streptomycin (P/S; Gibco, Thermo Fisher Scientific, Waltham, MA, USA) at 37 °C in 5% CO_2_ incubator. Caco-2 cells (1.34 × 10^5^ cells/cm^2^) were seeded on 12-well Transwell-Clear inserts with 0.4 μm pore polyester membranes and a membrane growth area of 1.12 cm^2^ (Corning), which were pre-coated with 5% Corning Matrigel matrix, LDEV-free (Corning). The culture medium was replaced every 2 days, and the cells were used for experiments after 2 weeks of cultivation.

hIEC progenitors were obtained from the Stem Cell Convergence Research Center, KRIBB (Daejeon, Korea) and were differentiated into hIECs following the protocol described by Kwon et al., 2021. The hIEC progenitors were seeded onto 1% Matrigel-coated tissue culture plate in hIEC differentiation medium 1 (hIEC medium 1) containing Dulbecco's Modified Eagle Medium/Nutrient Mixture F-12 (DMEM/F12; Gibco), epithelial growth factor (EGF) (100 ng/mL) (R&D Systems, Minneapolis, MN, USA), R-spondin1 (100 ng/mL) (Peprotech, Cranbury, NJ, USA), insulin (5 μg/mL) (Sigma-Aldrich, St. Louis, MO, USA), 2% FBS, 2% B27 supplement (Gibco), 1% N_2_ supplement (Gibco), 2 mM L-glutamine (Gibco), 1% non-essential amino acids (NEAAs; Gibco), 1% P/S and 15 mM Hepes buffer (Gibco). The hIEC medium 1 was changed every 2 days, and the hIEC progenitors were passaged once every 7 days. The hIEC progenitors cultured for 7 days were detached by treatment with trypsin-EDTA (Gibco) at 37 °C for 5 min and collected by centrifugation at 1,250 rpm for 5 min. The resulting cell pellet was resuspended, and 1.34 × 10^5^ cells/cm^2^ hIEC progenitors were reseeded onto 1% Matrigel-coated Transwell inserts with hIEC medium 1 containing 10 μM Y-27632 (Tocris Bioscience, Bristol, UK) and cultured for an additional 24 h. For differentiation into functional hIECs, the medium was replaced with hIEC differentiation medium 2 (hIEC medium 2) containing DMEM/F12, 2 μM Wnt-C59 (Selleck Chemicals, Houston, TX, USA), 100 ng/ml EGF, 1 mM valproic acid (Stemgent, Cambridge, MA, USA), 2% FBS, 1 % N_2_ supplement, 1 % B27 supplement, 2 mM L-glutamine, 1% NEAA, 1% P/S and 15 mM HEPES buffer. The hIEC medium 2 was replaced every 2 days, and the cells were cultured for 2 weeks prior to experiments.

### 2.3 Anaerobic co-culture of gut microbes with intestinal epithelial cell

After culturing the intestinal epithelial cells for 2 weeks as described above the medium in the basolateral compartment of the Transwell inserts was changed to antibiotic-free MEM containing 10% (v/v) FBS for Caco-2 cells or antibiotic-free hIEC medium 2 for hIECs. For anaerobic co-culture experiments, the cells were transferred to an anaerobic chamber. The apical medium was then replaced with deoxygenated medium comprised of 20% bacterial culture medium in cell culture medium. Overnight bacterial cultures were harvested by centrifugation, and the bacterial pellets were resuspended in the same apical co-culture medium at a volume equal to the original culture volume and adjusted to an OD_600_ of 1.0. The bacterial suspensions were then diluted as needed and inoculated into the apical compartment at 10^4^~10^6^ CFU/well for co-culture. The viable bacterial concentration in the inoculum was confirmed by serial dilution and plate counting at the start of co-culture.

### 2.4 Measurement of transepithelial electrical resistance (TEER)

TEER values of intestinal epithelial cell monolayers were measured at full confluence in the co-culture system using an epithelial tissue volt/ohmmeter (EVOM2, WPI, Sarasota, FL, USA) in accordance with the manufacturer's instructions.

### 2.5 Viability assay of intestinal epithelial cells

To evaluate the impact of anaerobic co-culture with bacteria on the viability of intestinal epithelial cells, the LIVE/DEAD™ viability/cytotoxicity kit (Thermo Fisher Scientific), containing calcein-AM to stain live cells and ethidium homodimer-1 to stain dead cells, was used according to the manufacturer's instructions. After staining the cells co-cultured with bacteria, live (green fluorescence) and dead (red fluorescence) cells were visualized using a fluorescence microscope. The resulting fluorescence images were quantified using ImageJ software.

### 2.6 Quantification of viable bacteria (CFU assay)

To assess bacterial growth, viable bacteria were quantified after co-culturing with intestinal epithelial cells for the designated time period. The apical medium (500 μL) from the Transwell insert was combined with 500 μL of 0.5% trypsin-EDTA used to detach bacteria adhered to the epithelial cells. The mixture was serially diluted and plated onto MRS or BHI agar, followed by incubation at 37 °C for 48 h under anaerobic conditions. The number of viable cells was measured by counting the colony-forming units (CFU). To compare bacterial growth during co-culture, growth kinetics were analyzed during the exponential phase. The specific growth rate (μ) was calculated using Equation 1,


$$\begin{array}{l} Growth\;rate\;\left(\mu \right)\;=\;\frac{\left(\ln\;X_{t}\;-\;\ln\;X_{0}\right)}{t} \end{array}$$ (1)

where *X*_0_ and *X*_*t*_ represent the bacterial counts (CFU/mL) at the beginning and end of the exponential phase, respectively, and t denotes the time interval.

The doubling time (dt) was calculated using Equation 2.


$$\begin{array}{l} Doubling\;time\;\left(dt\right)=\frac{ln2}{\mu } \end{array}$$ (2)

### 2.7 Scanning electron microscopy (SEM)

After co-culturing of intestinal epithelial cells with bacteria, cells in Transwell inserts were washed with PBS and fixed with 2.5% glutaraldehyde (Sigma-Aldrich) for primary fixation for 2 h at 4 °C. The cells were then washed with PBS, and the secondary fixation was carried out with osmium tetroxide (OsO_4_; Sigma-Aldrich) for 2 h at 4 °C. The fixed samples were washed with distilled water, and dehydration was performed through a graded ethanol series (30, 50, 70, 80, 90, and 100%; 5 min each). Hexamethyldisilazane (Sigma-Aldrich) was applied to the samples, which were then dried overnight in a fume hood at room temperature. Following excision from the inserts, the membranes were mounted on aluminum stubs and sputter-coated with platinum for 1 min using a CCU-010 coater (Safematic GmbH, Zizers, Switzerland). Images were acquired using a Regulus 8100 FE-SEM (Hitachi High-Tech, Tokyo, Japan).

### 2.8 Measurement of produced short-chain fatty acids (SCFAs)

To quantify the SCFAs produced during the co-culture, culture media from both the apical and basolateral compartments of the Transwell were collected separately. For the apical compartment, the supernatant was obtained by removing bacterial pellets through centrifugation, and both the apical supernatant and the basolateral medium were filtered prior to analysis. The amounts of produced SCFAs were determined using high-performance liquid chromatography (HPLC) system (Agilent 1200 Series, Agilent Technologies, Santa Clara, CA, USA) equipped with an Aminex^TM^ HPX-87H Organic Acid Column (300 × 7.8 mm, 9 μm particle size; Bio-Rad; Hercules, CA, USA) (Jo et al., 2024). For each metabolite, compartment-specific changes were calculated as the difference between the final and initial amounts in the apical and basolateral compartments. Relative basolateral change (%) was calculated using Equation 3,


$$\begin{array}{l} \begin{array}{l} Relative\;basolateral\;change\;\left(\%\right)= \end{array}\\ \frac{\left|\Delta basolateral\right|\;}{\left(\left|\Delta apical\right|+\;\left|\Delta basolateral\right|\right)}\times 100 \end{array}$$ (3)

where Δ represents the change in metabolite amount during the experimental period. This metric was used as a descriptive index of basolateral contribution to overall compartmental metabolite change and was not interpreted as a direct measure of SCFA permeability or transepithelial transport efficiency.

### 2.9 RNA extraction and quantitative real-time polymerase chain reaction

Total RNA was isolated from intestinal epithelial cells after co-culture with bacteria to assess changes in host gene expression. RNA extraction was performed using the Monarch Total RNA Miniprep Kit (New England Biolabs, Ipswich, MA, USA), and the concentration and purity of the RNA were assessed with a NanoDrop ND-1000 spectrophotometer (Thermo Scientific, Waltham, MA, USA). RNA samples with A260/A280 ratios within 1.9–2.1 and A260/A230 ratios ≥ 1.8 were used for downstream cDNA synthesis and qRT-PCR analysis. Subsequently, 1 μg of RNA was reverse-transcribed into cDNA using the iScript cDNA Synthesis Kit (Bio-Rad, Hercules, CA, USA). Quantitative real-time polymerase chain reaction (qRT-PCR) was performed using a CFX Connect Real-Time PCR System (Bio-Rad) with SsoAdvanced Universal SYBR Green Supermix (Bio-Rad). The cycling conditions were as follows: initial denaturation at 95 °C for 30 s, followed by 40 cycles of denaturation at 95 °C for 10 s and annealing/extension at 60 °C for 30 s. Melt curve analysis was performed to confirm amplification specificity. Relative gene expression levels were calculated using the 2^−Δ*ΔCT*^ method, and no additional fold-change threshold was applied for the targeted qRT-PCR. The primer sequences used in this study were obtained from a previous report (Kwon et al., 2021) and are listed in Supplementary Table 2.

### 2.10 Statistical analysis

All experiments were performed with at least three independent biological replicates, with technical replicates included when applicable and averaged prior to statistical analysis. Results are presented as means ± SD. Pairwise comparisons were performed using a two-tailed Student's *t*-test. For analyses involving multiple comparisons, *p-*values were adjusted using the Benjamini–Hochberg false discovery rate (FDR) method. Adjusted *p-*values < 0.05 were considered statistically significant.

## 3 Results

### 3.1 Design of an *in vitro* co-culture system for cultivating anaerobic gut microbes with intestinal epithelial cells

**Figure 1:**
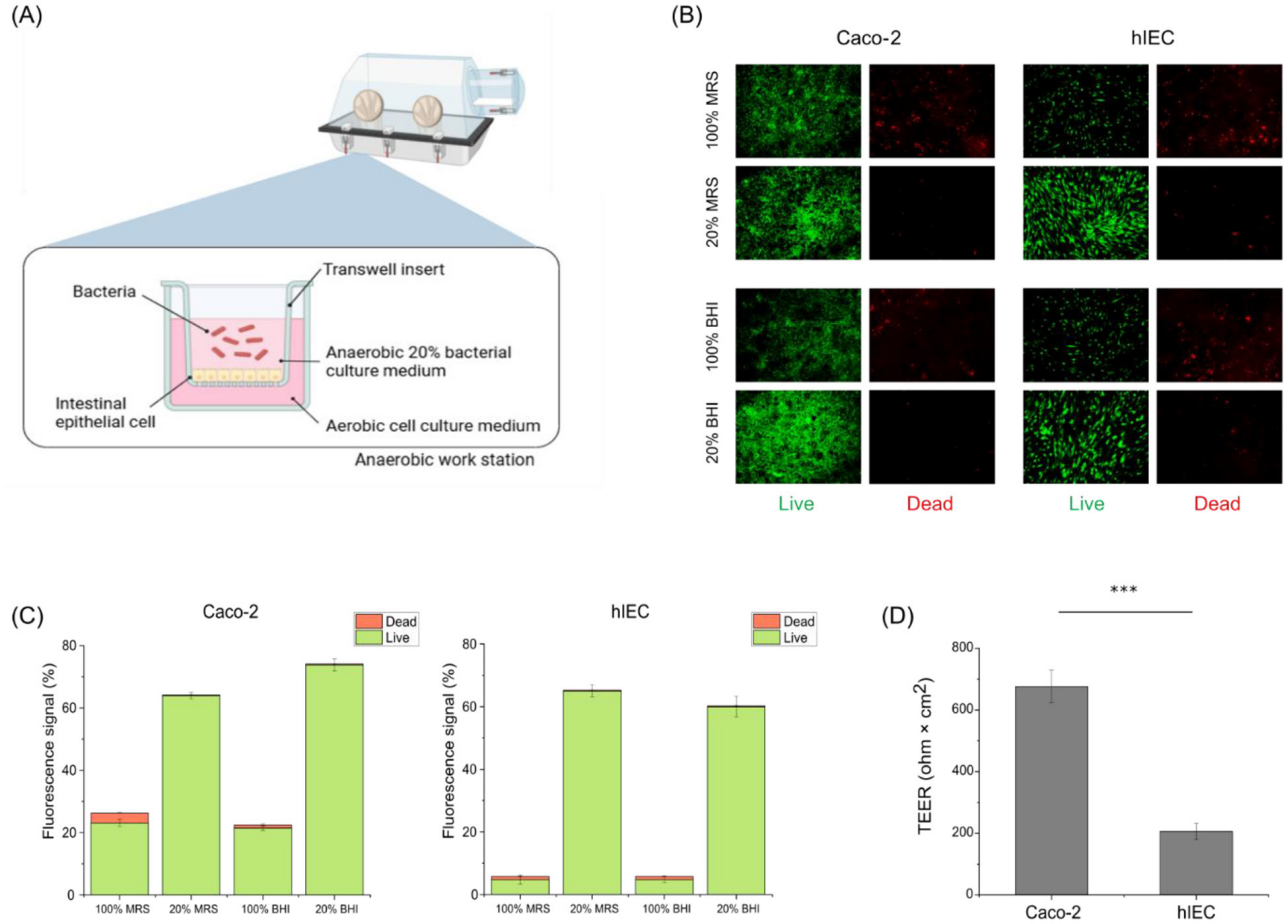
Development and validation of an anaerobic Transwell-based co-culture system for host-microbe interaction studies. **(A)** Schematic illustration of the co-culture system for cultivating anaerobic gut microbes with intestinal epithelial cells. Created with BioRender.com. **(B)** Viability of Caco-2 cells and hIECs after 12 h of culture in bacterial media at different dilution ratios. **(C)** Quantification of live and dead fluorescence signals (%) in Caco-2 cells and hIECs after 12 h of culture in bacterial media at different ratios. **(D)** TEER values of Caco-2 cell and hIEC monolayers. Data represent means ± SD (*n* = 6). ***p < 0.001 by a two-tailed Student's *t*-test.

A Transwell-based co-culture system was used to model anaerobic luminal exposure while maintaining intestinal epithelial monolayers on permeable inserts during the experimental period. Epithelial cells were grown as monolayers on insert membranes, and bacteria were introduced into the apical compartment. The Transwell assembly was placed inside an anaerobic chamber to support anaerobe cultivation, thereby maintaining oxygen-free conditions in the apical compartment. The basolateral compartment was supplied with oxygen-containing, antibiotics-free epithelial culture medium appropriate for each cell type (Figure 1A). Consistently, resazurin-based redox assessment confirmed that the apical compartment shifted toward and maintained a reduced redox environment, whereas the basolateral compartment retained a relatively more oxidized redox status during the co-culture period (Supplementary Figure 1).

We optimized apical medium conditions for epithelial survival during co-culture by assessing cell viability in Caco-2 cells and hIECs using bacterial media diluted in epithelial culture medium. Exposure to undiluted bacterial media (100% MRS or 100% BHI) reduced epithelial viability in both cell types, with extensive cell detachment and loss. In contrast, when bacterial media were diluted to 20% in epithelial culture medium, cell viability remained high for at least 12 h under both conditions (Figures 1B, C). Baseline TEER values, measured prior to bacterial exposure to confirm monolayer integrity, were 676.3 ± 57.4 Ω·cm^2^ and 206.5 ± 29.2 Ω·cm^2^ for Caco-2 cells and hIEC monolayers, respectively (Figure 1D), with the lower TEER of hIECs being closer to values reported for the human intestine (50–100 Ω·cm^2^) (Srinivasan et al., 2015).

Together, these results indicate that the optimized co-culture conditions support short-term maintenance of both epithelial cells and anaerobic gut bacteria and provide a defined platform for investigating host-microbe interactions under controlled anaerobic co-culture conditions. Subsequent co-culture experiments with anaerobic bacteria were then performed according to the workflow outlined in Supplementary Figure 2.

### 3.2 Co-culture of intestinal epithelial cells with *Lactiplantibacillus plantarum*

*L. plantarum* is a Gram-positive, facultatively anaerobic lactic acid bacterium belonging to the phylum *Bacillota*. Although many *L. plantarum* strains are associated with fermented foods and the human gastrointestinal tract, the WJL strain used in this study was originally isolated from the intestine of *Drosophila melanogaster* and was included as a host-associated *L. plantarum* comparator, consistent with its previous use in host physiology studies (Park et al., 2025). *L. plantarum* is known as a beneficial gut microorganism with antipathogenic bacterial and fungal properties, the ability to enhance gut barrier function (Liu et al., 2024), and the capacity to modulate inflammatory responses (Aljohani et al., 2025; Murofushi et al., 2015).

**Figure 2:**
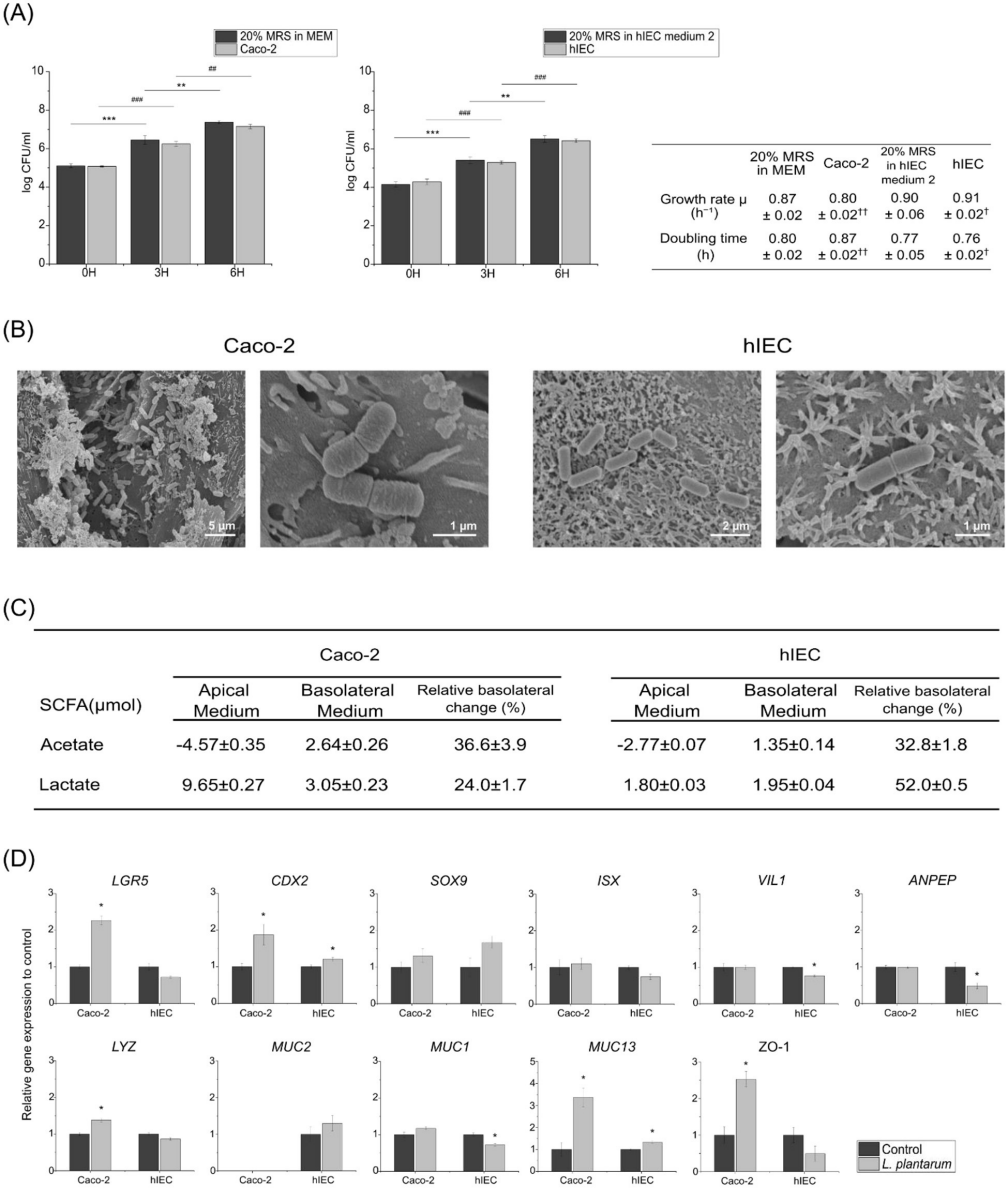
Co-culture of intestinal epithelial cells with *Lactiplantibacillus plantarum*. **(A)** Analysis of bacterial growth dynamics in co-culture with epithelial cells, revealing differences between Caco-2 cells and hIECs. In the CFU bar plots, bars represent mean log CFU/mL, and error bars indicate SD (*n* = 3). Asterisks indicate significant time-dependent changes in medium-only controls, whereas hash symbols indicate changes in epithelial co-culture conditions. Kinetic parameters are presented as means ± SD (*n* = 3) and were compared between each epithelial co-culture condition and its corresponding medium-only control.^†^*p* < 0.05; **, ^##^, or ^††^*p* < 0.01; ***, ^###^*p* < 0.001 by a two-tailed Student's *t*-test. **(B)** SEM images showing the surface association of *L. plantarum* with intestinal epithelial cells under co-culture conditions. **(C)** Changes in SCFA levels in apical and basolateral compartments, along with relative basolateral change. Data are presented as means ± SD (*n* = 3). Cross-model comparisons of absolute SCFA amounts should be interpreted cautiously because the basal media differed between the Caco-2 and hIEC systems. **(D)** Relative gene expression of intestinal markers in epithelial cells co-cultured with *L. plantarum* compared to control conditions. Data represent means ± SD (*n* = 3). Asterisks indicate significant differences relative to the corresponding untreated control within the same epithelial model (*FDR-adjusted *p* < 0.05).

*L. plantarum* was inoculated into the apical compartment of the co-culture system at an initial level of approximately 1.25 × 10^5^ CFU and cultured with intestinal epithelial cells for 6 h in an anaerobic chamber. This co-culture duration was selected to allow bacterial-epithelial interactions while maintaining epithelial cell viability (Supplementary Figure 3A). After 6 h of co-culture, *L. plantarum* growth was interpreted relative to each model's corresponding medium-only control. Growth in the hIEC co-culture remained comparable to its control, whereas co-culture with Caco-2 cells showed a reduced growth rate and prolonged doubling time (Figure 2A; Supplementary Table 3A). SEM analysis revealed distinct epithelial surface morphologies depending on the epithelial cell type. While Caco-2 cell monolayers displayed an uneven and rough surface upon co-culture with *L. plantarum*, hIEC monolayers maintained an intact microvillar structure, with *L. plantarum* localized in close proximity to the epithelial surface (Figure 2B).

In co-culture with *L. plantarum*, apical acetate levels decreased in both Caco-2 cells and hIECs, accompanied by detectable acetate changes in the basolateral compartment. Relative basolateral change values for acetate were 36.6% and 32.8% in Caco-2 cells and hIECs, respectively. Meanwhile, apical lactate levels increased in both Caco-2 cells and hIECs co-cultures, consistent with contributions from both bacterial and host cell metabolism, with a greater increase observed in Caco-2 cells (Figure 2C). Co-culture with *L. plantarum* differentially regulated intestinal marker expression, with significant changes in stem cell (*LGR5*), lineage-specific (*CDX2*), and membrane-associated mucin (*MUC13*) markers observed in Caco-2 cells (FDR-adjusted *p* < 0.05; Figure 2D). In contrast, hIECs exhibited an expression pattern that appeared relatively consistent with maintenance of a differentiated epithelial state. These results show distinct differences in *L. plantarum* growth, metabolite dynamics, and epithelial gene expression between Caco-2 cells and hIECs.

### 3.3 Co-culture of intestinal epithelial cells with *Bifidobacterium longum*

**Figure 3:**
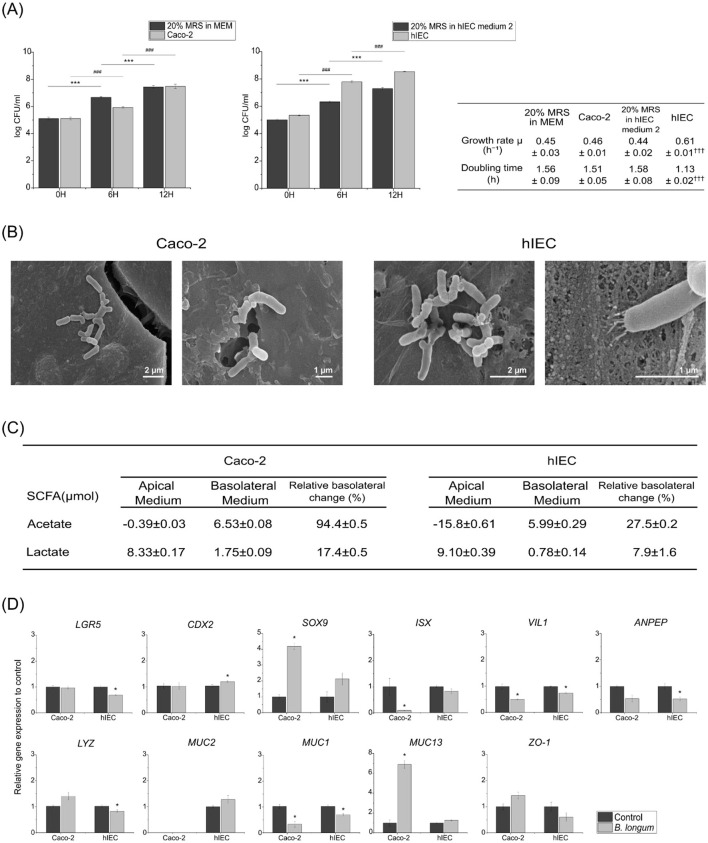
Co-culture of intestinal epithelial cells with *Bifidobacterium longum*. **(A)** Analysis of bacterial growth dynamics in co-culture with epithelial cells, revealing differences between Caco-2 cells and hIECs. In the CFU bar plots, bars represent mean log CFU/mL, and error bars indicate SD (*n* = 3). Asterisks indicate significant time-dependent changes in medium-only controls, whereas hash symbols indicate changes in epithelial co-culture conditions. Kinetic parameters are presented as means ± SD (*n* = 3) and were compared between each epithelial co-culture condition and its corresponding medium-only control. ***, ^###^, or ^†*††*^*p* < 0.001 by a two-tailed Student's *t*-test. **(B)** SEM images showing the surface association of *B. longum* with intestinal epithelial cells under co-culture conditions. **(C)** Changes in SCFA levels in apical and basolateral compartments, along with relative basolateral change. Data are presented as means ± SD (*n* = 3). Cross-model comparisons of absolute SCFA amounts should be interpreted cautiously because the basal media differed between the Caco-2 and hIEC systems. **(D)** Relative gene expression of intestinal markers in epithelial cells co-cultured with *B. longum* compared to control conditions. Data represent means ± SD (*n* = 3). Asterisks indicate significant differences relative to the corresponding untreated control within the same epithelial model (*FDR-adjusted *p* < 0.05).

*B. longum* is a Gram-positive, strictly anaerobic lactic acid bacterium belonging to the phylum *Actinobacteria* and a commensal species naturally inhabiting the human gastrointestinal tract (Kato et al., 2017). *B. longum* has been suggested to protect the intestinal mucosa against chronic inflammation and has been implicated in the prevention and treatment of gastrointestinal inflammatory diseases, including inflammatory bowel disease (Zhang et al., 2017; Yao et al., 2021). *B. longum* was inoculated into the apical compartment of co-culture system at an initial inoculum of approximately 1.47 × 10^5^ CFU and cultured with intestinal epithelial cells for 12 h, a duration selected to ensure sufficient bacterial expansion for growth kinetic analysis while maintaining epithelial integrity. No significant changes in epithelial cell viability were observed during the co-culture period (Supplementary Figure 3B). After 12 h of co-culture, *B. longum* expansion was evaluated relative to each corresponding medium-only control. The hIEC co-culture showed a marked increase over its hIEC medium 2-based control, whereas the Caco-2 co-culture showed only a modest increase over its MEM-based control (Figure 3A; Supplementary Table 3B). Bacterial association with Caco-2 cells appeared limited and less structured, whereas in hIECs, bacterial clusters were closely associated with densely developed microvilli, indicating a tighter spatial interaction (Figure 3B).

In co-culture with *B. longum*, substantial changes in acetate and lactate levels were observed in both the apical and basolateral compartments of Caco-2 and hIEC co-cultures. Differences in these compartmental SCFA changes may reflect model-dependent metabolite accumulation or distribution. Relative basolateral change values for acetate and lactate were 94.4% and 17.4% in Caco-2 cells and 27.5% and 7.9% in hIECs, respectively (Figure 3C). In terms of major intestinal cell type-associated markers, co-culture with *B. longum* elevated *SOX9* expression in Caco-2 cells (FDR-adjusted *p* < 0.05), with a similar directional increase observed in hIECs relative to their respective untreated controls, suggesting a stem/progenitor-associated response. In contrast, *VIL1* and *ANPEP* expression levels were decreased in both epithelial models relative to their respective untreated controls, with significant reductions observed particularly in hIECs (FDR-adjusted *p* < 0.05), suggesting a shift away from mature enterocyte-associated gene expression. Additionally, *B. longum* markedly induced *MUC13* expression in Caco-2 cells (FDR-adjusted *p* < 0.05), whereas hIECs showed minimal changes in mucin-related gene expression (Figure 3D). Together, these results indicate distinct differences in *B. longum* behavior and epithelial responses between the two co-culture models.

### 3.4 Co-culture of intestinal epithelial cells with *Akkermansia muciniphila*

**Figure 4:**
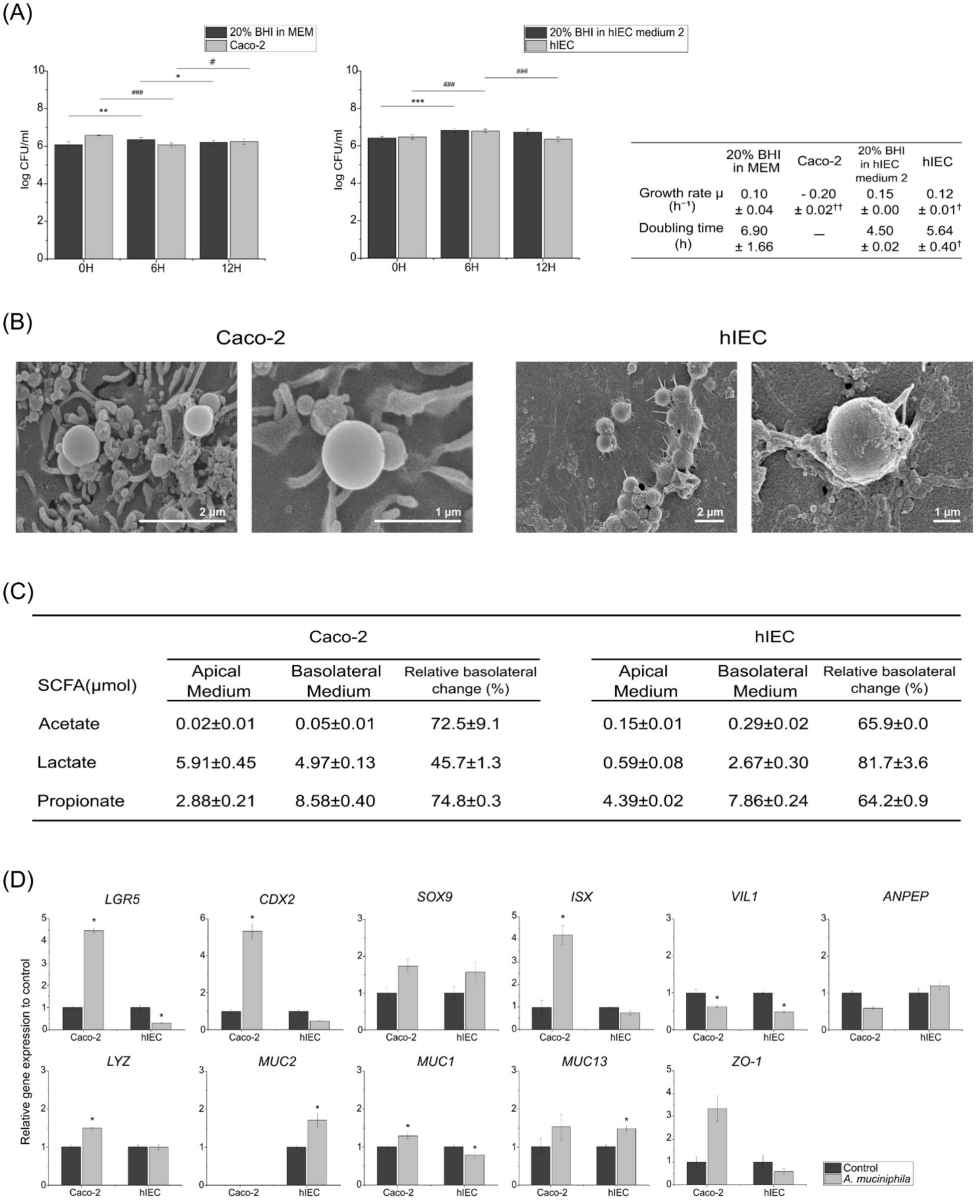
Co-culture of intestinal epithelial cells with *Akkermansia muciniphila*. **(A)** Analysis of bacterial growth dynamics in co-culture with epithelial cells, revealing differences between Caco-2 cells and hIECs. In the CFU bar plots, bars represent mean log CFU/mL, and error bars indicate SD (*n* = 3). Asterisks indicate significant time-dependent changes in medium-only controls, whereas hash symbols indicate changes in epithelial co-culture conditions. Kinetic parameters are presented as means ± SD (*n* = 3) and were compared between each epithelial co-culture condition and its corresponding medium-only control. *, ^#^, or ^†^*p* < 0.05; ** or ^††^*p* < 0.01; *** or ^###^, or ^†*††*^*p* < 0.001 by a two-tailed Student's *t*-test. **(B)** SEM images showing the surface association of *A. muciniphila* with intestinal epithelial cells under co-culture conditions. **(C)** Changes in SCFA levels in apical and basolateral compartments, along with relative basolateral change. Data are presented as means ± SD (*n* = 3). Cross-model comparisons of absolute SCFA amounts should be interpreted cautiously because the basal media differed between the Caco-2 and hIEC systems. **(D)** Relative gene expression of intestinal markers in epithelial cells co-cultured with *A. muciniphila* compared to control conditions. Data represent means ± SD (*n* = 3). Asterisks indicate significant differences relative to the corresponding untreated control within the same epithelial model (*FDR-adjusted *p* < 0.05).

*A. muciniphila* is a Gram-negative, strictly anaerobic bacterium belonging to the phylum *Verrucomicrobia* and is known for its capacity to degrade intestinal mucin (Derrien et al., 2004). It has been reported to ameliorate metabolic syndrome and intestinal mucosal injury by promoting anti-inflammatory responses and contributing to intestinal homeostasis (Rodrigues et al., 2022; Zhao et al., 2024). *A. muciniphila* was introduced into the apical compartment of the co-culture system at an initial level of approximately 2.67 × 10^6^ CFU and incubated with intestinal epithelial cells for 12 h, a duration that did not significantly affect epithelial cell viability (Supplementary Figure 3C). During the 12-h co-culture period, *A. muciniphila* growth was interpreted relative to each corresponding medium-only control. Although proliferation remained lower than the control in both models, the hIEC co-culture retained a positive growth rate, whereas bacterial counts declined from the initial inoculum in the Caco-2 model (Figure 4A; Supplementary Table 3C). Bacterial attachment appeared limited in Caco-2 co-culture, whereas in the hIEC co-culture, clustered bacterial populations were observed in close contact with the epithelial surface, occasionally surrounded by extracellular material (Figure 4B).

Co-culture with *A. muciniphila* resulted in detectable SCFA changes in both epithelial models (Figure 4C). Acetate and propionate showed high relative basolateral change values in Caco-2 cells (71.4% and 74.9%, respectively) and hIECs (65.9% and 64.2%), consistent with compartmental redistribution across the epithelial monolayer rather than direct measurement of transport efficiency. In contrast, lactate displayed model-dependent behavior, with higher accumulation and moderate relative basolateral change values in Caco-2 cells. Gene expression changes also displayed model-dependent patterns in the two co-culture systems (Figure 4D). *A. muciniphila* significantly increased the expression of intestinal transcription factors (*CDX2* and *ISX*) and the intestinal stem cell marker (*LGR5*) in Caco-2 cells (FDR-adjusted *p* < 0.05). In addition, *A. muciniphila* upregulated *MUC2* gene expression in hIECs (FDR-adjusted *p* < 0.05). Collectively, these findings show model-dependent variation in *A. muciniphila* distribution, metabolite profiles, and epithelial responses.

### 3.5 Co-culture of intestinal epithelial cells with *Clostridioides difficile*

**Figure 5:**
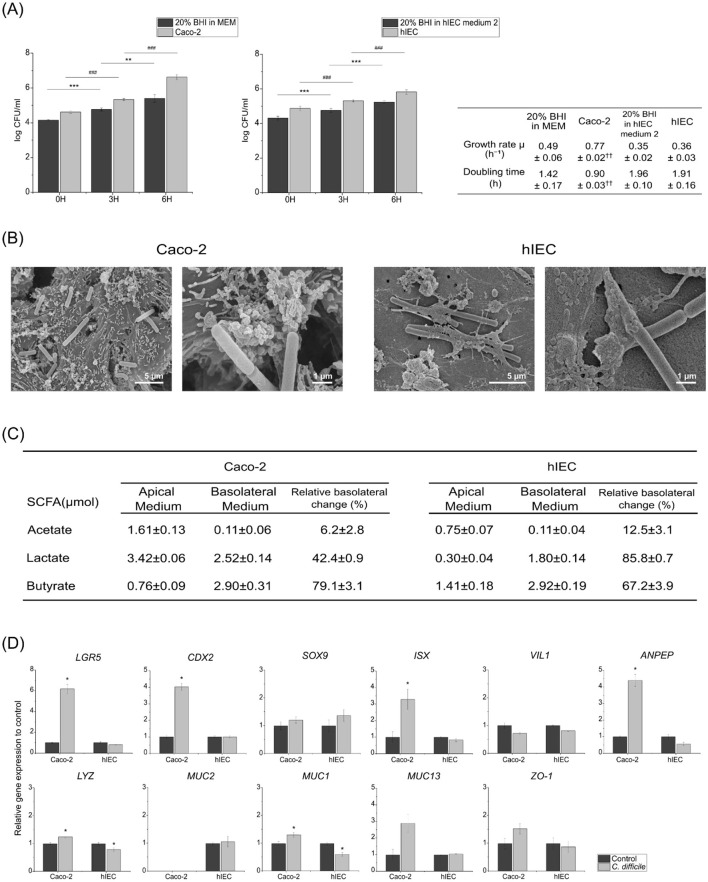
Co-culture of intestinal epithelial cells with *Clostridioides difficile*. **(A)** Analysis of bacterial growth dynamics in co-culture with epithelial cells, revealing differences between Caco-2 cells and hIECs. In the CFU bar plots, bars represent mean log CFU/mL, and error bars indicate SD (*n* = 3). Asterisks indicate significant time-dependent changes in medium-only controls, whereas hash symbols indicate changes in epithelial co-culture conditions. Kinetic parameters are presented as means ± SD (*n* = 3) and were compared between each epithelial co-culture condition and its corresponding medium-only control. ** or ^††^*p* < 0.01; *** or ^###^*p* < 0.001 by a two-tailed Student's t-test. **(B)** SEM images showing the surface association of *C. difficile* with intestinal epithelial cells under co-culture conditions. **(C)** Changes in SCFA levels in apical and basolateral compartments, along with relative basolateral change. Data are presented as means ± SD (*n* = 3). Cross-model comparisons of absolute SCFA amounts should be interpreted cautiously because the basal media differed between the Caco-2 and hIEC systems. **(D)** Relative gene expression of intestinal markers in epithelial cells co-cultured with *C. difficile* compared to control conditions. Data represent means ± SD (*n* = 3). Asterisks indicate significant differences relative to the corresponding untreated control within the same epithelial model (*FDR-adjusted *p* < 0.05).

*C. difficile* is a Gram-positive, obligate anaerobic, spore-forming bacterium belonging to the phylum *Bacillota*. It is an opportunistic intestinal pathogen that can cause severe and potentially life-threatening diarrhea (Sandhu and McBride, 2018). A hallmark of its pathogenesis is spore formation, which enables persistence, transmission, and resistance to antibiotics and environmental insults (Lee et al., 2022). *C. difficile* was applied to the apical compartment of the co-culture system at an initial level of approximately 3.57 × 10^4^ CFU and maintained with intestinal epithelial cells for 6 h, a duration that did not significantly compromise epithelial viability (Supplementary Figure 3D). Relative to the corresponding medium-only controls, *C. difficile* showed a more pronounced growth response in the Caco-2 co-culture than in the hIEC co-culture, as reflected by the growth-kinetic parameters (Figure 5A; Supplementary Table 3D). SEM analysis further demonstrated model-dependent differences in bacterial distribution (Figure 5B). Bacteria were positioned above the epithelial surface with limited direct contact in Caco-2 cells, whereas in hIECs they were more frequently observed in close apposition to the epithelial interface.

During co-culture with *C. difficile*, acetate, lactate, and butyrate changes were detected, consistent with central metabolic and organic acid fermentation pathways reported for this bacterium (Neumann-Schaal et al., 2019; Figure 5C). Butyrate showed substantial relative basolateral change values in both epithelial models, reaching 79.2% in Caco-2 cells and 67.4% in hIECs. With respect to gene expression in intestinal epithelial cells, intestinal transcription factors (*CDX2* and *ISX*), the stem cell marker *LGR5*, and the enterocyte-associated marker *ANPEP* were markedly upregulated in Caco-2 cells upon co-culture with *C. difficile* (FDR-adjusted *p* < 0.05), whereas such changes were not observed in hIECs (Figure 5D). These results reveal clear differences in *C. difficile* growth dynamics, metabolite levels, and epithelial gene expression between the two models.

### 3.6 Co-culture of intestinal epithelial cells with *Escherichia coli*

**Figure 6:**
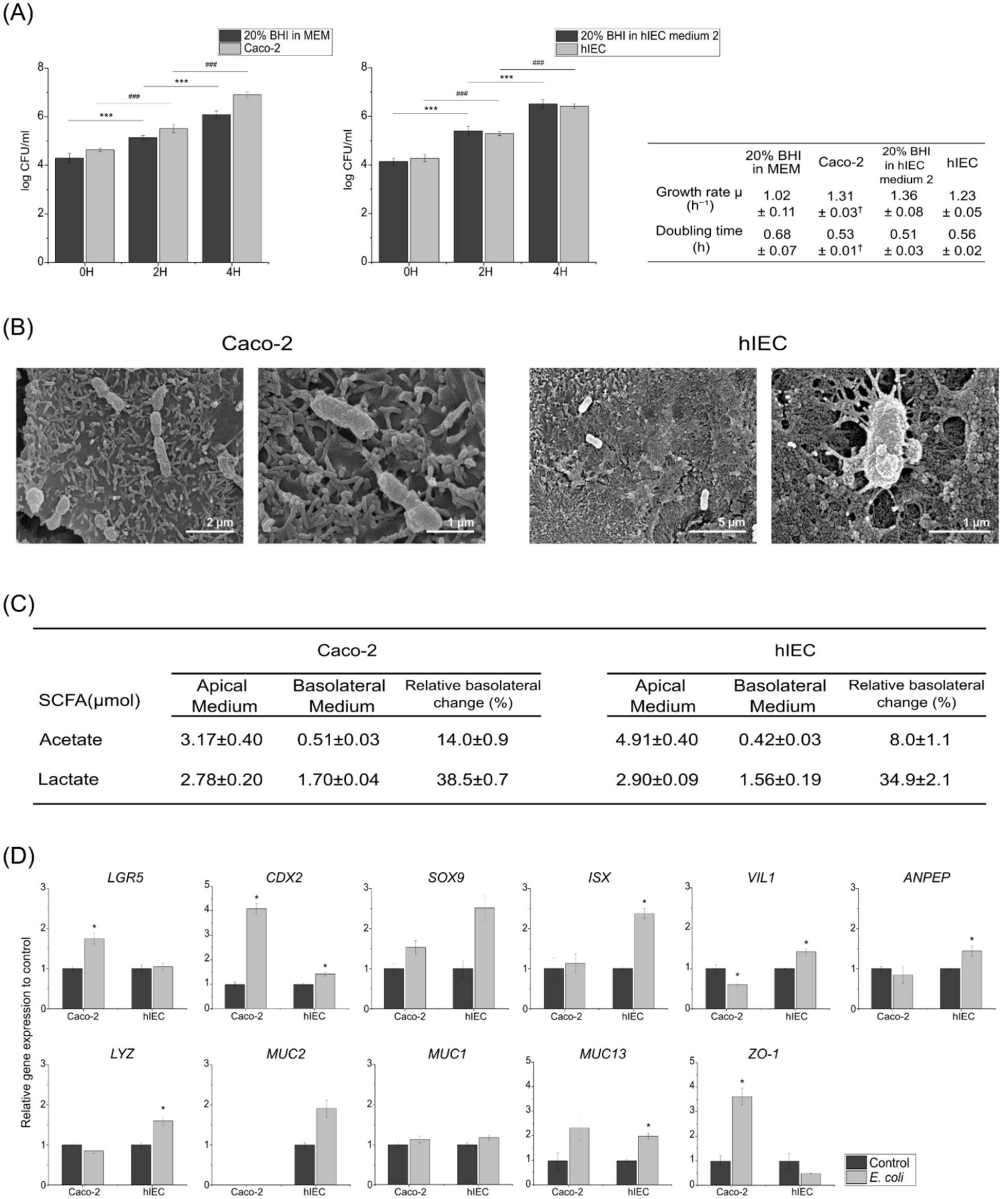
Co-culture of intestinal epithelial cells with *Escherichia coli*. **(A)** Analysis of bacterial growth dynamics in co-culture with epithelial cells, revealing differences between Caco-2 cells and hIECs. In the CFU bar plots, bars represent mean log CFU/mL, and error bars indicate SD (*n* = 3). Asterisks indicate significant time-dependent changes in medium-only controls, whereas hash symbols indicate changes in epithelial co-culture conditions. Kinetic parameters are presented as means ± SD (*n* = 3) and were compared between each epithelial co-culture condition and its corresponding medium-only control. ^†^*p* < 0.05; *** or ^###^ *p* < 0.001 by a two-tailed Student's t-test. **(B)** SEM images showing the surface association of *E. coli* with intestinal epithelial cells under co-culture conditions. **(C)** Changes in SCFA levels in apical and basolateral compartments, along with relative basolateral change. Data are presented as means ± SD (*n* = 3). Cross-model comparisons of absolute SCFA amounts should be interpreted cautiously because the basal media differed between the Caco-2 and hIEC systems. **(D)** Relative gene expression of intestinal markers in epithelial cells co-cultured with *E. coli* compared to control conditions. Data represent means ± SD (*n* = 3). Asterisks indicate significant differences relative to the corresponding untreated control within the same epithelial model (*FDR-adjusted *p* < 0.05).

*E. coli* is a Gram-negative, facultative anaerobic bacterium belonging to phylum *Pseudomonadota*. Based on distinct virulence determinants and pathogenic features, *E. coli* strains are classified into commensal, intestinal pathogen (diarrheagenic), and extraintestinal pathogenic types, which are associated with abdominal infections, urinary tract infections, sepsis, and meningitis (Wang et al., 2013). *E. coli* was introduced into the apical side of the co-culture system at an initial level of approximately 2.42 × 10^4^ CFU and co-cultured with intestinal epithelial cells for 4 h. Compared with each corresponding medium-only control, *E. coli* growth was enhanced in the Caco-2 co-culture, whereas growth in the hIEC co-culture remained broadly comparable to its hIEC medium 2-based control during the 4-h incubation period (Figure 6A; Supplementary Table 3E). Notably, co-culture with *E. coli* for 4 h resulted in an increase in dead cells in the Caco-2 model, but not in hIECs (Supplementary Figure 3E). SEM analysis revealed model-dependent differences in bacterial localization. *E. coli* was observed among microvillus-like projections in Caco-2 cells, whereas in hIECs it exhibited greater surface association with epithelial surface (Figure 6B).

During the co-culture, acetate and lactate were detected in both epithelial models (Figure 6C). Acetate accumulation was observed in both epithelial co-culture conditions, with different apical and basolateral change patterns between the models. Relative basolateral change values for acetate were 13.8% in Caco-2 cells and 7.9% in hIECs. For lactate, relative basolateral change values were 38.5% in Caco-2 cells and 34.9% in hIECs. In addition, *E. coli* induced the expression of intestinal transcription factor *CDX2* and stem cell-associated marker *LGR5* in Caco-2 cells, and *ISX* in hIECs (FDR-adjusted *p* < 0.05). Also, in hIECs, *E. coli* co-culture increased the expression of enterocyte-associated markers (*VIL1* and *ANPEP*), secretory lineage marker *LYZ*, and the mature intestinal marker *MUC13* (FDR-adjusted *p* < 0.05; Figure 6D). Collectively, these findings demonstrate that *E. coli* induces epithelial model-dependent differences in growth, metabolite production, and host responses, with hIECs exhibiting a more differentiated and potentially adaptive epithelial response.

## 4 Discussion

In this study, we used an accessible anaerobic Transwell-based co-culture system that enables the co-maintenance of oxygen-sensitive bacteria and oxygen-dependent intestinal epithelial cells in a conventional and accessible format. Rather than presenting the Transwell implementation itself as the principal advance, our study focuses on its use for a systematic, side-by-side comparison of tumor-derived Caco-2 cells and stem cell-derived hIECs under comparable anaerobic co-culture conditions. Using this platform, we found that epithelial model selection substantially altered microbial proliferation, metabolite distribution, and host transcriptional responses. These results indicate that epithelial cell origin is not a passive experimental variable but a determinant of host-microbe interaction readouts *in vitro*.

A general pattern emerged across multiple bacterial species. When interpreted relative to each model's corresponding medium-only control, hIECs tended to better support the growth or persistence of beneficial and commensal anaerobes, including *L. plantarum, B. longum*, and *A. muciniphila*, whereas Caco-2 cells showed stronger growth responses for opportunistic bacteria such as *C. difficile* and *E. coli*. Because these comparisons were performed within an anaerobic Transwell co-culture framework across multiple bacterial species, they extend previous apical anaerobic or Transwell-based co-culture studies by emphasizing how epithelial model choice can change bacterial growth, metabolite profiles, and host transcriptional responses. These findings suggest that epithelial cell origin can influence microbial ecological outcomes *in vitro*. Because hIECs more closely recapitulate intestinal lineage features, including mucus-associated epithelial properties, they may provide a luminal environment that differs from Caco-2 cells and may better approximate the healthy intestinal niche.

Species-specific responses further reinforce this interpretation. The relatively greater growth or persistence of *A. muciniphila* in hIECs may be partly related to mucus-associated features of stem cell-derived epithelial models, given the mucin-utilizing capacity of this bacterium. Previous characterization of the hIEC model demonstrated MUC2-positive goblet-like cells (Kwon et al., 2021), and in the present study *A. muciniphila* induced *MUC2* mRNA expression in hIECs, consistent with previous reports showing that *A. muciniphila*-derived products can enhance goblet cell-associated *MUC2* expression (Tingler and Engevik, 2025). However, because mucus layer thickness, Alcian blue/PAS staining, and MUC2 protein levels were not quantified in the present study, direct evidence for mucus-mediated support remains lacking. In contrast, the strong proliferation of *C. difficile* in Caco-2 cells may reflect a microenvironment that favors opportunistic pathogens, potentially linked to differences in nutrient availability and epithelial metabolic state. The upregulation of *ANPEP* expression in Caco-2 cells during *C. difficile* co-culture may also contribute to a local environment that supports bacterial growth, although this possibility will require further mechanistic validation.

Differences in SCFA profiles and relative basolateral appearance also highlighted the influence of epithelial model choice on metabolite readouts. Given that measured SCFA levels arise from a combination of bacterial production, host cell metabolism, epithelial uptake, and transepithelial transport, the observed differences between Caco-2 cells and hIECs suggest that metabolite data depend on the epithelial model used rather than reflecting bacterial activity alone. In particular, the elevated lactate levels observed in several Caco-2 co-cultures may be consistent with altered tumor-cell metabolism, including enhanced glycolytic lactate production, but this interpretation remains speculative without direct metabolic-flux analysis (Liberti and Locasale, 2016). Similarly, differences in basolateral appearance across multiple SCFAs may indicate model-dependent variation in epithelial handling or retention. However, because Caco-2 cells and hIECs were maintained in different basal epithelial media during co-culture, cross-model comparisons of absolute metabolite amounts were interpreted cautiously and in relation to the corresponding medium-only controls.

Furthermore, because absolute metabolite concentrations can also be influenced by bacterial abundance, we performed an exploratory CFU area under the curve (CFU AUC)-normalized analysis to evaluate metabolite accumulation relative to the time-integrated bacterial load (Supplementary Figure 4). Across the five bacterial species tested, CFU AUC-normalized metabolite patterns did not simply mirror bacterial growth patterns, suggesting that metabolite profiles were not explained by bacterial abundance alone. However, because metabolite levels in epithelial co-culture may also be affected by epithelial uptake, metabolism, retention, and transepithelial transport, these normalized values can be interpreted as metabolite accumulation relative to bacterial load rather than direct per-cell bacterial production rates.

The transcriptional data support the importance of epithelial context. In Caco-2 cells, microbial exposure frequently triggered broad induction of intestinal transcription factors, stem cell-associated markers, and the tight junction protein ZO-1. While these responses may reflect active host signaling, they may also indicate model-specific stress, altered differentiation dynamics, or a transformed metabolic state that differs from that of normal intestinal epithelium. In contrast, hIECs exhibited more selective and lineage-consistent transcriptional responses under the defined hIEC culture conditions compared to Caco-2 cells. Notably, co-culture with *E. coli* was associated with increased expression of enterocyte-associated markers, secretory lineage markers, and the mature intestinal marker *MUC13*, suggesting model-context-dependent transcriptional changes related to epithelial differentiation and maturation.

A further limitation is that hIECs were maintained in hIEC differentiation medium 2, which contains Wnt-C59, valproic acid, and EGF to preserve functional epithelial characteristics. These components can modulate pathways associated with epithelial stemness, differentiation, and lineage specification, which overlap with several markers assessed in this study, including *LGR5, SOX9, CDX2*, and lineage-associated genes. Therefore, the transcriptional differences observed between Caco-2 cells and hIECs should be interpreted as epithelial model-context-dependent responses rather than solely as cell-intrinsic differences. Future studies using medium-matched conditions, modulator withdrawal, or individual modulator controls will be needed to separate epithelial cell type-specific effects from culture-medium-dependent effects.

The co-culture duration was set to a relatively short period of up to 12 h to maintain epithelial viability while enabling measurable bacterial and metabolite responses. Because the bacterial species differed in growth characteristics, epithelial compatibility, and cytotoxic potential, strain-specific inoculum concentrations and exposure durations were selected based on preliminary optimization experiments. Therefore, absolute CFU values and metabolite levels were not interpreted as direct quantitative comparisons across bacterial species, but were evaluated primarily within each species relative to the corresponding medium-only controls. Importantly, epithelial viability was preserved under these conditions, supporting the maintenance of oxygen-dependent epithelial cells within the co-culture system. Although resazurin-based assessment provided information on the redox status of the culture compartments, further characterization of oxygen/redox gradients, together with post-treatment TEER or permeability measurements, would help refine the physiological and mechanistic interpretation of the model. In addition, several experimental factors should be considered when interpreting the model-dependent differences observed in this study. First, although the mucus-associated phenotype of hIECs and the induction of *MUC2* mRNA by *A. muciniphila* support a possible mucus-related explanation, we did not directly quantify mucus layer thickness, Alcian blue/PAS staining, or MUC2 protein levels. Therefore, the contribution of mucus to bacterial support should be interpreted as a hypothesis requiring further validation. Second, Caco-2 cells and hIECs required different epithelial basal media during co-culture; thus, direct comparisons of raw CFU values or absolute metabolite levels between the two models may reflect both epithelial cell type and basal-medium effects. Third, gene-expression analysis was normalized using a single reference gene, *GAPDH*, which may limit the quantitative robustness of transcriptional comparisons. Future studies incorporating multiple validated reference genes and additional protein-level or functional readouts will further strengthen interpretation of epithelial lineage and differentiation responses. Finally, although hIEC TEER values were closer to the reported human intestinal range than those of Caco-2 cells, they remained higher than the *in vivo* range cited in this study. Thus, hIECs should be considered a complementary epithelial model with improved lineage features rather than a fully physiological or universally superior substitute for native intestinal epithelium. Despite these limitations, our findings provide a practical framework for improving the physiological relevance of *in vitro* gut co-culture models. We demonstrate that epithelial cell origin can fundamentally shape microbial behavior, metabolite dynamics, and host responses. These findings have important implications for studies aiming to compare bacterial fitness, characterize metabolite exchange, or interpret host responses with translational relevance.

Our results demonstrate that epithelial cell origin substantially shapes microbial behavior, metabolite dynamics, and host responses *in vitro*. Taken together, these findings highlight the limitations of tumor-derived epithelial cell lines and support the inclusion of hIEC-based systems as complementary, lineage-rich epithelial models that can reveal context-dependent host–microbe responses not fully captured by Caco-2 cells. At the same time, our data support the conclusion that epithelial model choice changes experimental outcomes, rather than establishing hIECs as universally superior for all applications. Accordingly, systematic side-by-side comparisons under defined co-culture conditions may help improve interpretation of bacterial fitness, metabolite exchange, and host epithelial responses *in vitro*.

## Data Availability

The original contributions presented in the study are included in the article/Supplementary material, further inquiries can be directed to the corresponding author.
