# Supplementary material for: Epithelial model-dependent host-microbe interactions in human intestinal models under anaerobic Transwell co-culture
Source: Front Microbiol. 2026 Jul 24;17:1886960. doi: 10.3389/fmicb.2026.1886960 (PMC13447449; doi:10.3389/fmicb.2026.1886960)
Supplement: Supplementary file 1 [file Data_Sheet_1.docx]

Supplementary Material

# Supplementary Figures and Tables

| **Species** | **Strain** | **Isolation source** |
| --- | --- | --- |
| *Lactiplantibacillus plantarum* | WJL | *Drosophila melanogaster* intestine |
| *Bifidobacterium longum* subsp*. longum* | BP1883457* | Human oral cavity |
| *Akkermansia muciniphila* | KCTC15667^T^ | Human intestinal tract (feces) |
| *Clostridioides difficile* | BP1429630* | Human feces |
| *Escherichia coli* | KCTC2571 | Feces |

**Supplementary Table 1.** The bacterial strains used in this study.

***Strains are R&D bioresources deposited by Doo-Sang Park (https://biorp.kribb.re.kr).

**Supplementary Table 2.** Primer sets used for quantitative real-time polymerase chain reaction (qRT-PCR).

| Gene | Primer (Forward) | Primer (Reverse) |
| --- | --- | --- |
| *GAPDH* | GAAGGTGAAGGTCGGAGTC | GAAGATGGTGATGGGATTTC |
| *LGR5* | TGCTCTTCACCAACTGCATC | CTCAGGCTCACCAGATCCTC |
| *CDX2* | CTGGAGCTGGAGAAGGAGTTTC | ATTTTAACCTGCCTCTCAGAGAGC |
| *SOX9* | GGAGAGCGAGGAGGACAAGTTC | TTGAAGATGGCGTTGGGGG |
| *ISX* | CAGGAAGGAAGGAAGAGCAA | TGGGTAGTGGGTAAAGTGGAA |
| *VIL1* | AGCCAGATCACTGCTGAGGT | TGGACAGGTGTTCCTCCTTC |
| *ANPEP* | AAGCCTGTTTCCTCGTTGTC | AACCTCATCCAGGCAGTGAC |
| *LYZ* | AAAACCCCAGGAGCAGTTAAT | CAACCCTCTTTGCACAAGCT |
| *MUC2* | TGTAGGCATCGCTCTTCTCA | GACACCATCTACCTCACCCG |
| *MUC1* | CTTCCTGCTGCTGCTCCTCAC | TCTCTGGGTAGCCGAAGTCTCC |
| *MUC13* | CGGATGACTGCCTCAATGGT | AAAGACGCTCCCTTCTGCTC |
| *ZO-1* | TGTGAGTCCTTCAGCTGTGGAA | GGAACTCAACACACCATTG |

**Supplementary Table 3.** CFU counts across media conditions and epithelial models. Values are presented as mean ± SD CFU/well from three biological replicates.

**(A) *L. plantarum***

| **Time (h)** | **20% MRS in MEM** | **Caco-2** | **20% MRS in hIEC medium 2** | **hIEC** |
| --- | --- | --- | --- | --- |
| 0 | (1.28 ± 0.09) × 10⁵ | (1.20 ± 0.01) × 10⁵ | (1.23 ± 0.25) × 10⁵ | (1.28 ± 0.09) × 10⁵ |
| 3 | (2.94 ± 0.98) × 10⁶ | (1.77 ± 0.22) × 10⁶ | (1.71 ± 0.13) × 10⁶ | (3.49 ± 0.23) × 10⁶ |
| 6 | (2.37 ± 0.10) × 10⁷ | (1.43 ± 0.13) × 10⁷ | (2.75 ± 0.44) × 10⁷ | (3.04 ± 0.13) × 10⁷ |

**(B) *B. longum***

| **Time (h)** | **20% MRS in MEM** | **Caco-2** | **20% MRS in hIEC medium 2** | **hIEC** |
| --- | --- | --- | --- | --- |
| 0 | (1.30 ± 0.26) × 10⁵ | (1.30 ± 0.26) × 10⁵ | (1.03 ± 0.09) × 10⁵ | (2.25 ± 0.25) × 10⁵ |
| 6 | (4.74 ± 0.50) × 10⁶ | (8.50 ± 1.15) × 10⁵ | (2.12 ± 0.26) × 10⁶ | (6.12 ± 0.10) × 10⁷ |
| 12 | (2.77 ± 0.63) × 10⁷ | (3.17 ± 1.13) × 10⁷ | (2.00 ± 0.35) × 10⁷ | (3.46 ± 0.13) × 10⁸ |

**(C) *A. muciniphila***

| **Time (h)** | **20% BHI in MEM** | **Caco-2** | **20% BHI in hIEC medium 2** | **hIEC** |
| --- | --- | --- | --- | --- |
| 0 | (1.20 ± 0.27) × 10⁶ | (3.80 ± 0.02) × 10⁶ | (2.65 ± 0.12) × 10⁶ | (3.03 ± 0.29) × 10⁶ |
| 6 | (2.23 ± 0.17) × 10⁶ | (1.15 ± 0.11) × 10⁶ | (6.71 ± 0.37) × 10⁶ | (6.35 ± 0.35) × 10⁶ |
| 12 | (1.60 ± 0.16) × 10⁶ | (1.77 ± 0.27) × 10⁶ | (5.48 ± 1.08) × 10⁶ | (2.28 ± 0.21) × 10⁶ |

**(D) *C. difficile***

| **Time (h)** | **20% BHI in MEM** | **Caco-2** | **20% BHI in hIEC medium 2** | **hIEC** |
| --- | --- | --- | --- | --- |
| 0 | (1.41 ± 0.01) × 10⁴ | (3.95 ± 0.20) × 10⁴ | (2.06 ± 0.16) × 10⁴ | (6.87 ± 0.55) × 10⁴ |
| 3 | (5.87 ± 0.30) × 10⁴ | (2.15 ± 0.05) × 10⁵ | (5.65 ± 0.59) × 10⁴ | (2.05 ± 0.05) × 10⁵ |
| 6 | (2.63 ± 0.95) × 10⁵ | (4.28 ± 0.69) × 10⁶ | (1.71 ± 0.06) × 10⁵ | (6.67 ± 0.71) × 10⁵ |

**(E) *E. coli***

| **Time (h)** | **20% BHI in MEM** | **Caco-2** | **20% BHI in hIEC medium 2** | **hIEC** |
| --- | --- | --- | --- | --- |
| 0 | (2.03 ± 0.50) × 10⁴ | (4.32 ± 0.16) × 10⁴ | (1.40 ± 0.17) × 10⁴ | (1.92 ± 0.30) × 10⁴ |
| 2 | (1.39 ± 0.06) × 10⁵ | (3.32 ± 0.83) × 10⁵ | (2.60 ± 0.53) × 10⁵ | (1.96 ± 0.09) × 10⁵ |
| 4 | (1.22 ± 0.20) × 10⁶ | (8.09 ± 0.70) × 10⁶ | (3.30 ± 0.62) × 10⁶ | (2.64 ± 0.16) × 10⁶ |

**Supplementary Figure 1.** Resazurin-based characterization of redox status in the anaerobic Transwell co-culture system. Blank-corrected absorbance at 570 and 600 nm was measured in the apical and basolateral compartments of Caco-2 and hIEC co-culture systems. Resazurin was added at a final concentration of 1 mg/L, and absorbance values were corrected by subtracting matched no-resazurin controls for each compartment and time point. Data are shown as mean ± SD. The apical compartment showed a rapid loss of A600 signal relative to A570, indicating rapid reduction of resazurin and suggesting that the apical compartment rapidly shifted to and maintained a strongly reduced redox environment. In contrast, the basolateral compartment showed relatively greater retention of the A600 signal, suggesting that although partial reduction occurred over time, it maintained a relatively more oxidized redox status than the apical compartment during co-culture.

**Supplementary Method**

Resazurin was used to assess the redox status of the Transwell co-culture system. Resazurin stock solution was added to the apical and basolateral compartments at a final concentration of 1 mg/L, and absorbance was measured at 570 and 600 nm at the indicated time points. Matched no-resazurin controls were included for each compartment and time point, and absorbance values were corrected by subtracting the corresponding control values.

**Supplementary Figure 2.** Experimental workflow of the anaerobic host-microbe co-culture system.

**Supplementary Figure 3.** Epithelial cell viability during co-culture with bacteria.

**Supplementary Figure 4.** CFU area under the curve (CFU AUC)-normalized metabolite changes during epithelial co-culture. Metabolite changes in the apical and basolateral compartments were normalized to the time-integrated bacterial load, calculated as CFU AUC, and are expressed as metabolite change per 10⁸ CFU·h. Asterisks above hIEC bars indicate significant differences between Caco-2 and hIEC within the same metabolite and compartment (*FDR-adjusted p < 0.05, two-tailed Student’s t-test followed by Benjamini–Hochberg FDR correction).
